# Near-Wall Aggregation of Amyloidogenic Aβ 1-40 Peptide: Direct Observation by the FRET

**DOI:** 10.3390/molecules26247590

**Published:** 2021-12-15

**Authors:** Natalia Katina, Alisa Mikhaylina, Nelly Ilina, Irina Eliseeva, Vitalii Balobanov

**Affiliations:** Institute of Protein Research, Pushchino, 142290 Moscow, Russia; nkatina@phys.protres.ru (N.K.); alisamikhaylina15@gmail.com (A.M.); nelly.ilyina@mail.ru (N.I.); yeliseeva@vega.protres.ru (I.E.)

**Keywords:** amyloidogenesis, aggregation, adsorption, Aβ 1-40 peptide, boundary of liquid phase

## Abstract

The formation of amyloid fibrils is one of the variants of the self-organization of polypeptide chains. For the amyloid aggregation, the solution must be oversaturated with proteins. The interface of the liquid (solution) and solid (vessel walls) phases can trigger the adsorption of protein molecules, and the resulting oversaturation can initiate conformational transitions in them. In any laboratory experiment, we cannot exclude the presence of surfaces such as the walls of vessels, cuvettes, etc. However, in many works devoted to the study of amyloid formation, this feature is not considered. In our work, we investigated the behavior of the Aβ 1-40 peptide at the water–glass, water–quartz, and water–plastic interface. We carried out a series of simple experiments and showed that the Aβ 1-40 peptide is actively adsorbed on these surfaces, which leads to a significant interaction and aggregation of peptides. This means that the interface can be the place where the first amyloid nucleus appears. We suggest that this effect may also be one of the reasons for the difficulty of reproducing kinetic data when studying the aggregation of the amyloid of the Aβ 1-40 peptide and other amyloidogenic proteins

## 1. Introduction

Amyloid aggregation remains one of the most exciting areas in the biophysics of protein molecules. Great efforts have been directed to studying the first stages of aggregation—the formation of the nucleus of amyloid fibrils. Many researchers suggest their spontaneous appearance in solution [1,2]. This interpretation draws an analogy between the process of amyloid aggregation and the process of crystallization. Goto also draws an analogy between amyloid formation and crystallization in his recent works [3]. As is known, under natural conditions, crystallization begins at inhomogeneities—dust grains, surface defects, etc. The rate of crystallization on such inhomogeneities significantly exceeds the rate of spontaneous crystallization from solution [4,5]. Studies devoted to the effect of various surfaces on amyloid formation also indicate similar phenomena during the initiation of the amyloid fibrils growth [6,7,8]. This behavior is typical for nucleation and elongation systems. The speed of the whole process depends on a random event—the appearance of the first seed of a new phase [9]. This makes the process difficult to experimentally reproduce. Poor reproducibility of the results is one of the complex problems in the experimental study of the kinetics of amyloid fibril formation. The search for a solution to this problem led us to two possible explanations. The first follows from the theory of nucleation and elongation—these are just the nature of the aggregation process. That is, a single random event triggers the aggregation. Second, there is a systematic factor that is not taken into account by researchers. Instead, these two explanations are combined into one: an unaccounted factor leads to the appearance of seeds.

The area of interest of the presented work was the effect of the surface of the vessel, cuvette, or test tube, in which amyloid aggregates are formed. This interest is easy to explain. On the one hand, papers indicate that the surface can modulate the aggregation process [10,11]; on the other hand, in most works, the question of the surface effect influence on the process in bulk is omitted [12]. To investigate the processes occurring on the glass surface, in the conditions of our experiments, studies of the aggregation of Aβ 1-40 peptides were carried out. We labeled Aβ 1-40 peptides by fluorescent dyes Cy5 and Cy3 and used them for observation. We have shown that Aβ 1-40 peptide is adsorbed in significant amounts on the glass surface, which leads to the significant interaction and aggregation of peptides.

## 2. Results and Discussion

### 2.1. The Choice of the Method of Observation

A number of spectral and microscopic methods can be used to monitor protein aggregation and amyloid formation [10]. Each of them has its area of application, advantages, and disadvantages. For our research, the possibility of direct observation without the stage of sample preparation is essential. In AFM and TEM, a small portion of the solution is taken as a sample, limiting the field of view. In addition, fixation of the sample to the base sheet can lead to artifacts. The SPR method requires fixing one of the interacting components on a previously prepared substrate. Having analyzed the available research methods in this way, we concluded that fluorescent methods are the most acceptable. Additionally, since there are no tryptophan residues in the Aβ peptide 1-40 sequence, we need an added fluorescent label. The most commonly used label for amylolide structures is the ThioflavinT dye. However, it does not fully satisfy our needs, since it has a low binding to early-stage aggregation species compared to mature fibrils. Namely, these stages are the most interesting and least studied.

**Figure 1:**
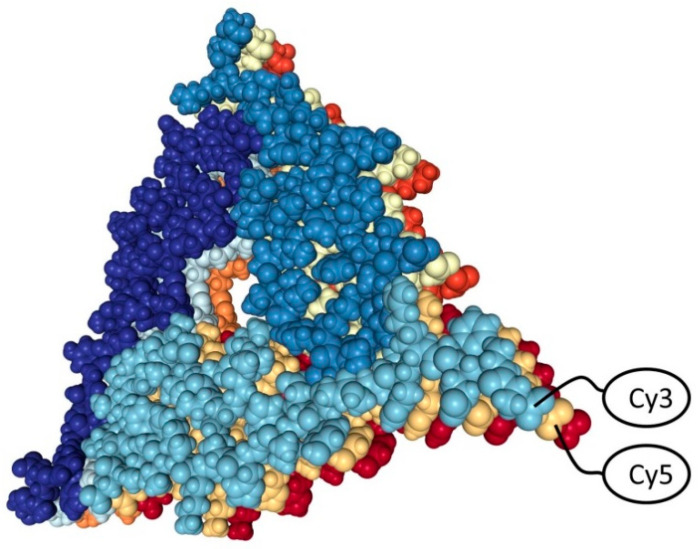
Structure of Aβ 1-40 amyloid fibril (PDB: 2M4J). Fluorescent dyes Cy3 and Cy5 attached to the N terminus of peptides are schematically indicated.

So, we chose the fluorescence of the attached dyes as the observation method. In this case, we used two dyes that form a FRET pair—Cy3 and Cy5. When these dyes become closer fluorescence energy of Cy3 can transfer to Cy5. This effect allows to evaluate the interaction between peptides. According to the data of a structural study, in the amyloid fibril of Aβ peptide 1–40, the peptides are located so that their C-terminus is covered from the solvent, and the N-terminus are relatively accessible and close one by one (see Figure 1) [13]. Therefore, we assumed that the peptides marked on the N-terminus would bring the dyes closer together during the formation of amyloid structures, which we will register by the change in the fluorescence spectrum. It was planned in this way to record the kinetics of the initial stages of aggregation.

### 2.2. FRET Measurement in Bulk Solution and on Surfaces

**Figure 2:**
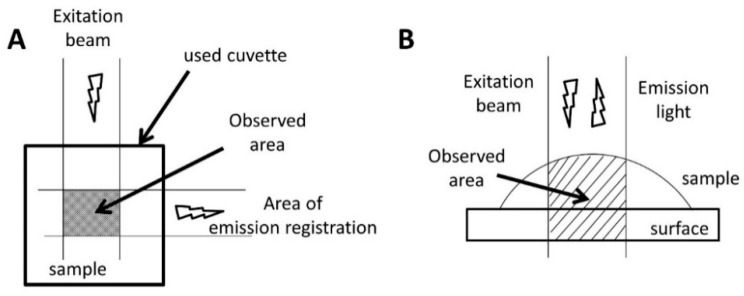
Scheme of fluorescence research in the standard version (**A**) and the fluorescence study on the surface (**B**). Again, the observed areas are shaded.

One-half of the peptides were labeled Cy3 dye, which served as a fluorescence donor. The other half was marked with Cy5 dye, which served as an acceptor. The experiments described below were used such peptides in a 1:1 ratio, mixed before or during the experiment. The first experiments carried out in the bulk volume of a solution did not give the desired results. At the initial moments, the FRET signal was not registered. Further research shows that this can be explained by the fact that the objects of our interest were not located in the observed area. We schematically illustrated this idea in Figure 2A. As can be seen, when using the classical design of the fluorescence experiment, only the signal in crossing the excitation light beam and the detector’s field of view can be registered. The walls of the cuvette are not into this area; therefore, it is not possible to observe what is happening on them.

**Figure 3:**
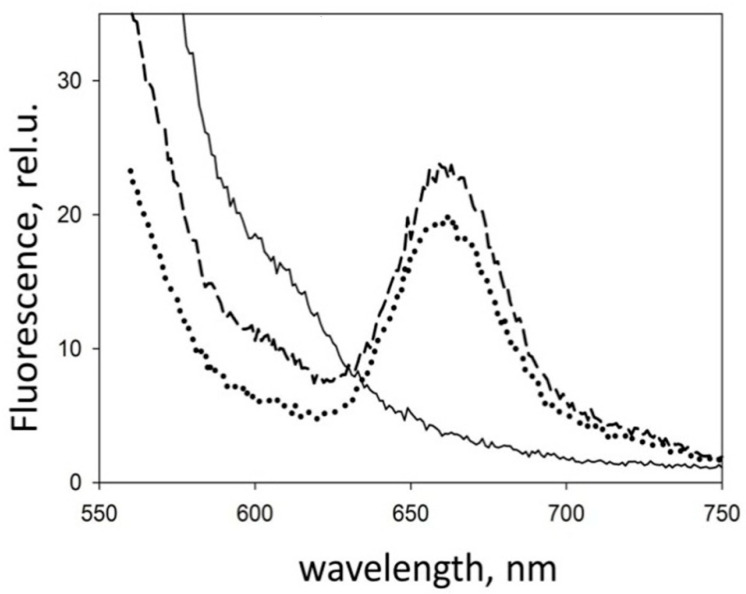
Fluorescence of a droplet of a peptide solution on the glass surface with excitation at 513 nm. A peptide labeled only with Cy3 is a solid line. A mixture of peptides marked Cy3 and Cy5 is a dashed line. The fluorescence of a glass surface gently washed after measuring the mix of peptides is a dotted line.

To test our hypothesis, we changed the design of the experiment. Fluorescence at the interface now also fell into the field of view (Figure 2B). First, the fluorescence of a droplet of a peptide solution labeled with Cy3 dye was measured on the glass surface. Then, a solution of the peptide labeled with the Cy5 dye was added to it. In this case, a fluorescence peak of Cy5 appeared, which indicates the interaction of peptides labeled with different dyes. After that, we gently washed the glass surface and applied a drop of the buffer solution without peptides. The spectrum retained the Cy5 fluorescence peak. The results present in Figure 3 show that the peptides were adsorbed on the surface and were not washed off. This result redirected our experiments towards finding and refining the site of initial aggregation of Aβ 1-40 peptides.

Since the study of amyloid aggregation is carried out not only in glass containers but also in plastic test tubes and in quartz cuvettes, we investigated the influence of these surfaces as well. We obtained similar results. The appearance of FRET is also observed on these surfaces. This signal also remains after flushing the surface.

A reasonable question arises: are the attached dyes the driving force behind the sorption of peptides on the surface? We carried out an additional experiment, which showed that dyes Cy3 and Cy5 by themselves in concentrations comparable to those used in our experiments are not adsorbed in significant amounts on the glass surface. This indicates that they are not the driving force behind peptide–surface interactions. In what follows, we proceeded from the assumption that the observed effects are a consequence of the properties of the peptides.

### 2.3. Visualization of the Aggregation Site Using Confocal Fluorescence Microscopy

**Figure 4:**
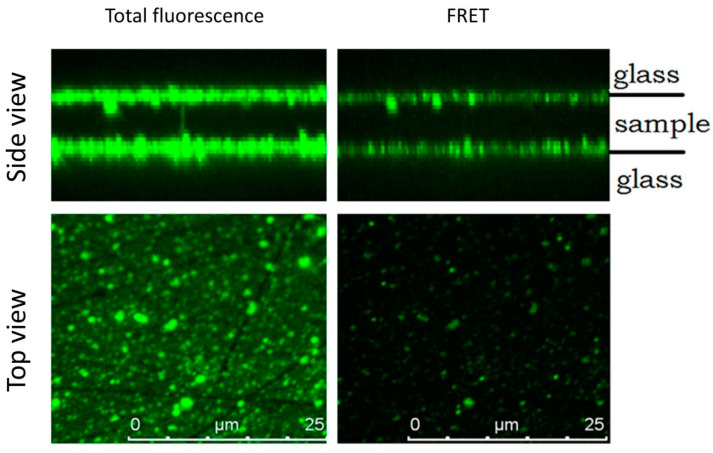
Fluorescent confocal microscopy images. The top view and reconstruction of the side view in the channels of general fluorescence (excitation at 532 nm, emission registration at 533–778 nm) and the FRET fluorescence (excitation at 532 nm emission registration at 650–778 nm) are shown.

To clarify and visualize the localization of interacting peptides, we used a confocal fluorescence microscope. Solution of mixed peptides was applied onto the microscope slides and covered with a coverslip slide. The entire space was examined layer by layer, from the slide to the coverslip, capturing their surfaces. In the channel of general fluorescence (excitation and registration of fluorescence of both dyes), we found that most fluorescent particles are concentrated on glass surfaces (see Figure 4). Moreover, a significant transfer of fluorescent energy (excitation of Cy3 fluorescence and registration of Cy5 fluorescence) is observed precisely on the glass surface. This fact indicates the interface as a possible place for a significant increase in the concentration of peptides and, consequently, acceleration of their interaction and aggregation.

### 2.4. Can We Avoid Surface Effects?

**Figure 5:**
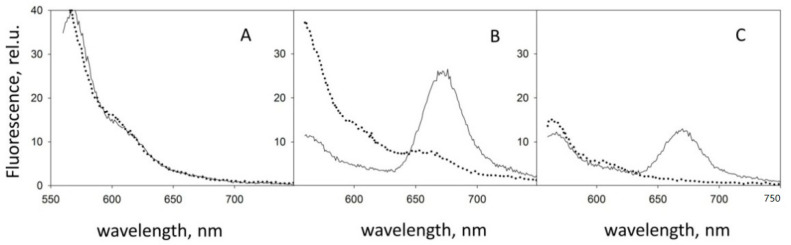
The results of FRET experiments on pure glass (solid lines) and glass surfaces treated by BSA (dotted lines). (**A**) A peptide labeled with Cy3 only; (**B**) a peptide labeled with Cy5 is added; (**C**) fluorescence of the glass surface gently washed with a buffer after measuring the mixture.

The next logical step in this research is to find ways to inhibit surface effects. To do this, we need to change the properties of the surface and reduce its ability to adsorb protein. Without inventing a new one, we used a well-proven way with preliminary processing of the glass surface with a BSA solution. The experiments described in Section 2.2 were repeated. The results of this experiment are shown in Figure 5. On the surface treated with BSA, the effect of fluorescent energy transfer was almost completely suppressed. That means no close interaction between peptides without accessible glass surfaces. No peptide adsorption was observed on the treated surface. The absence of adsorption indicates the absence of binding of the Aβ to BSA located on the surface. In conjunction with the fact that the BSA does not go from the surface into the solution (we checked this spectrophotometrically), we can conclude that the reason for the change in behavior is precisely in the hiding of the glass surface from peptides.

This experiment also answered the critical question: does the interaction occur between already sorbed proteins, or does aggregation occur first, and then adsorption? Since no energy transfer is observed when glass surfaces are inaccessible, the interaction between peptides occurs only on the accessible surface already after their adsorption and not in the bulk solution. In addition, since there is no significant increase in FRET during the experiment with a treated surface, the rate of aggregation of peptides in bulk solution is much lower. This fact indicates the surface as a factor of aggregation accelerating.

### 2.5. Conclusions

The analysis of the presented results concludes that it is necessary to take into account the phenomena occurring with proteins on the surface of the vessels in which the experiment is carried out, especially when it happens by concentration-dependent nucleation and elongation as in the case of aggregation and amyloid formation. Any vessel used in an experiment has walls that can affect protein adsorption and change the protein structure. The critical question: what is the influence of surface effects on the behavior of the protein in bulk? However, answering this question remains outside the scope of this article. This question is especially acute in the presence of solution movement, for example, when pouring or stirring. Undoubtedly, it should be the subject of further research.

The influence of various surfaces on amyloid aggregation has been studied for a long time. However, as the results of this work show, this factor can take place in any experiment without the addition of specific surfaces. The interface between the liquid phase and the vessel wall is sufficient. As the analysis of the literature has shown, this fact falls out of the field of view of researchers almost always when surface effects are not an object of interest. From our point of view, the described effects are pretty suitable for the role of unaccounted systemic factors affecting the reproducibility of experiments on the study of amyloid aggregation. Projecting this thought onto living organisms, we see a variety of different surfaces, each of which can influence the process of amyloid formation in its way. However, it is worth noting that there should not be so many surfaces that provoke amyloid formation; otherwise, amyloidosis would be much more frequent.

## 3. Materials and Methods

### 3.1. Gene Expression; Isolation and Purification of Aβ Peptide 1-40

The plasmid vector pET-32 LIC allows one to express the target peptide gene fused with the trx gene of thioredoxin within a hybrid protein. The hybrid protein also carries the 6×His sequence to ensure rapid and efficient purification of the product using metal chelate chromatography. The expression level of hybrid protein in this construct was appreciably high, making it possible to produce more target proteins. The expression of genes was performed in E. coli strain BL-21 DE3. Once expression had been completed, E. coli cells were precipitated by centrifugation at 6000× *g* and 4 °C for 15 min.

The resulting cell biomass was resuspended in 50 mM Tris-HCl, pH 8.0, 1 mM EDTA, 0.02 M β-mercaptoethanol; MgCl_2_ (to a final concentration of 5 mM) and DNase I (Sigma-Aldrich, St. Louis, MO, USA) (50 µg per g of cells) were added. The cell suspension was homogenized on a French press (Spectronic Instruments, Inc., Irvine, CA, USA). The water-insoluble fraction of the homogenate was precipitated by centrifugation at 30,000 rpm for 30 min; approximately 90% of hybrid protein was present in the water-soluble fraction. The hybrid protein was isolated from the cell lysate by ion-exchange chromatography on a DEAE-cellulose column (Sigma-Aldrich, St. Louis, MO, USA) in 20 mM Tris-HCl, pH 8.0, 100 mM NaCl. The hybrid protein was eluted with 50–500 mM NaCl gradient in the same buffer.

Next, the hybrid protein was purified on a column packed with nickel-nitrilotriacetic acid (Ni-NTA)metal chelate resin (Qiagen, Hilden, Germany) equilibrated with 50 mM Tris-HCl buffer, pH 8.0, containing 300 mM NaCl. The hybrid protein was eluted with 20–250 mM imidazole gradient. The purified hybrid protein was concentrated on the DEAE-cellulose column until a concentration of at least 10 mg/mL. The hybrid protein was subjected to enzymatic cleavage using factor Xa protease (Sigma-Aldrich, St. Louis, MO, USA) in 20 mM Tris-HCl buffer, pH 8.0, containing 100 mM NaCl and 5 mM CaCl_2_, at room temperature for 24 h at a 1:2000 molar ratio between the enzyme and the protein. The reaction mixture was applied to the column packed with Ni-NTA in the respective buffer; the target Aβ peptide 1-40 was not bound to the sorbent and was eluted from the column immediately after the void volume mark. The purity and homogeneity of the obtained peptide was assessed by electrophoresis in 15% SDS-PAAG.

### 3.2. Peptide Labeling

To obtain fluorescently labeled peptides, we used fluorescent dyes manufactured by Jena Bioscience Gmbh (Jena, Germany). The peptides were chemically bound to Cy3 Cy5 dyes according to the manufacturer’s protocol. The pH of the solution was 7, following the manufacturer’s recommendations for selective binding to the N-terminus of peptides. Separation of labeled peptides and unbound dye was performed by SEC on a Sephadex G-25 column. The labeling efficiency was assessed spectrophotometrically in accordance with the manufacturer’s guidelines.

### 3.3. Fluorescence Spectroscopy

The fluorescence measurements were made on a Cary Eclipse fluorescence spectrophotometer (Varian, Palo Alto, CA, USA). Measurements were taken in a quartz cuvette 3 × 3 mm for a standard variant and used a plate reader to measure the fluorescence on the surface. The fluorescence spectra were recorded at 550–750 nm at an excitation wavelength of 513 nm. The final concentration of the peptide was 0.03 mg/mL.

### 3.4. Confocal Fluorescence Microscopy

A Leica TCS SPE confocal fluorescent microscope was used to clarify and visualize the localization of interacting peptides. The specimens (5 µL) were applied onto the microscope slides and covered with a coverslip slide. Confocal fluorescence microscopy images were recorded with excitation at 532 and emission registration at 553–778 nm for general fluorescence and at 650–778 nm for FRET. The final concentration of the peptide was 0.03 mg/mL.

## Data Availability

Not applicable.

## Sample Availability

Samples of the compounds are not available from the authors.
